# Supplementary material for: A Ribosome Interaction Surface Sensitive to mRNA GCN Periodicity
Source: Biomolecules. 2020 Jun 3;10(6):849. doi: 10.3390/biom10060849 (PMC7357141; doi:10.3390/biom10060849)
Supplement: Supplementary file 1 [file biomolecules-10-00849-s001.zip › supporting_final/FigS2.pdf]

H-bonds over time (ns)

Stage I

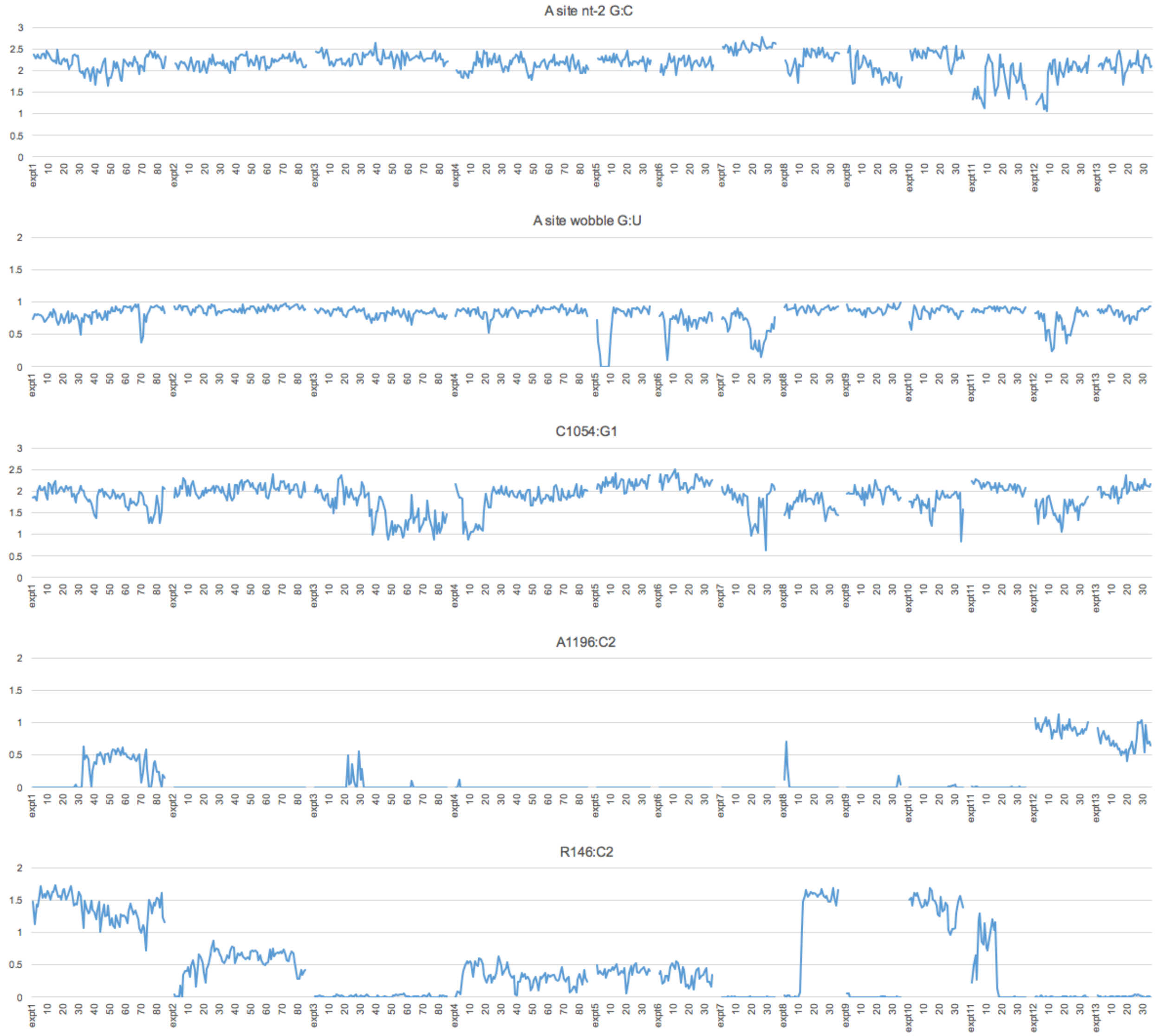

Figure S2

H-bonds over time (ns) cont.

Stage II

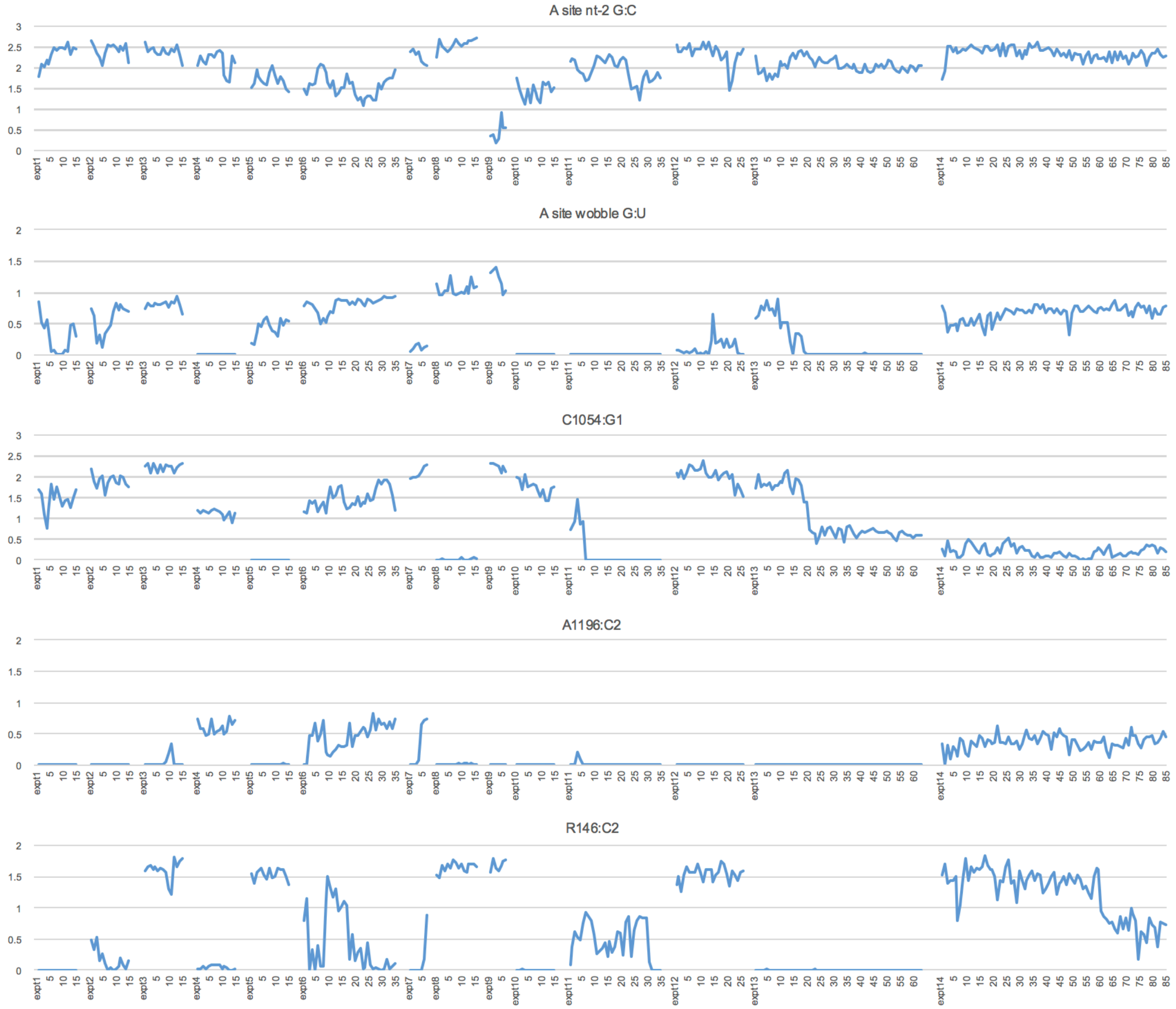

H-bonds over time (ns) cont.

Stage III

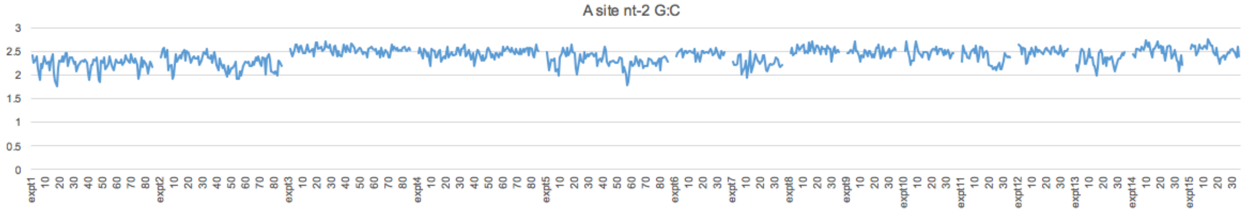

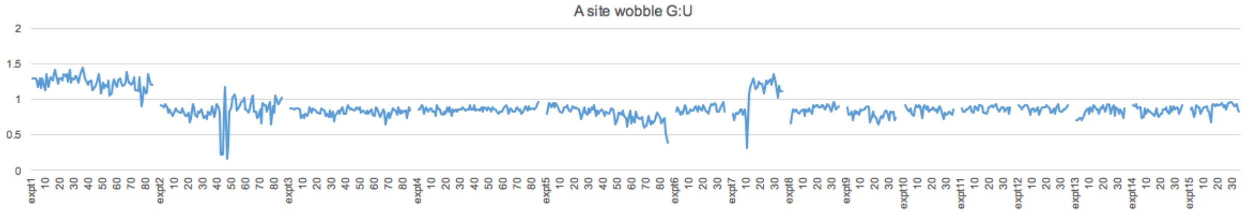

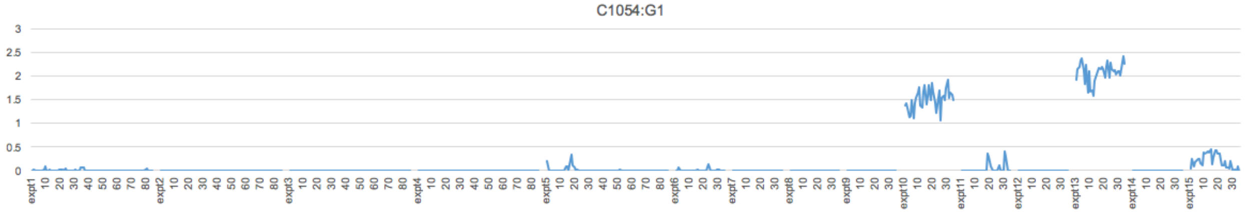

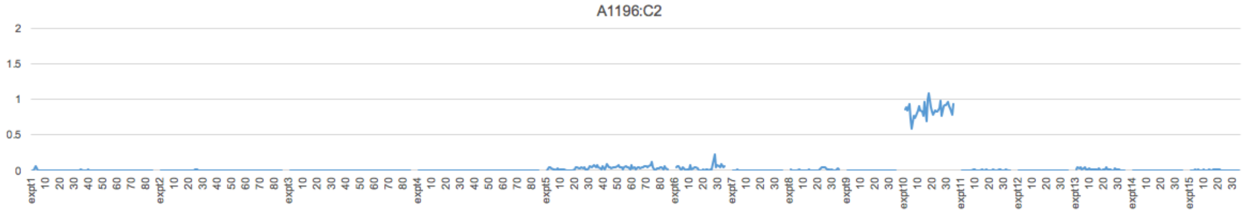

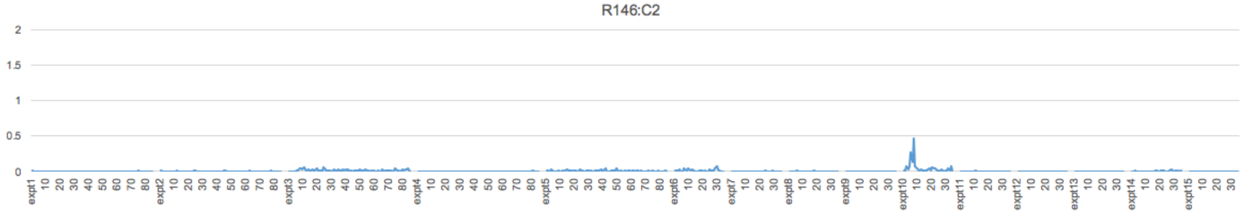

H-bonds over time (ns) cont.

Stage IV

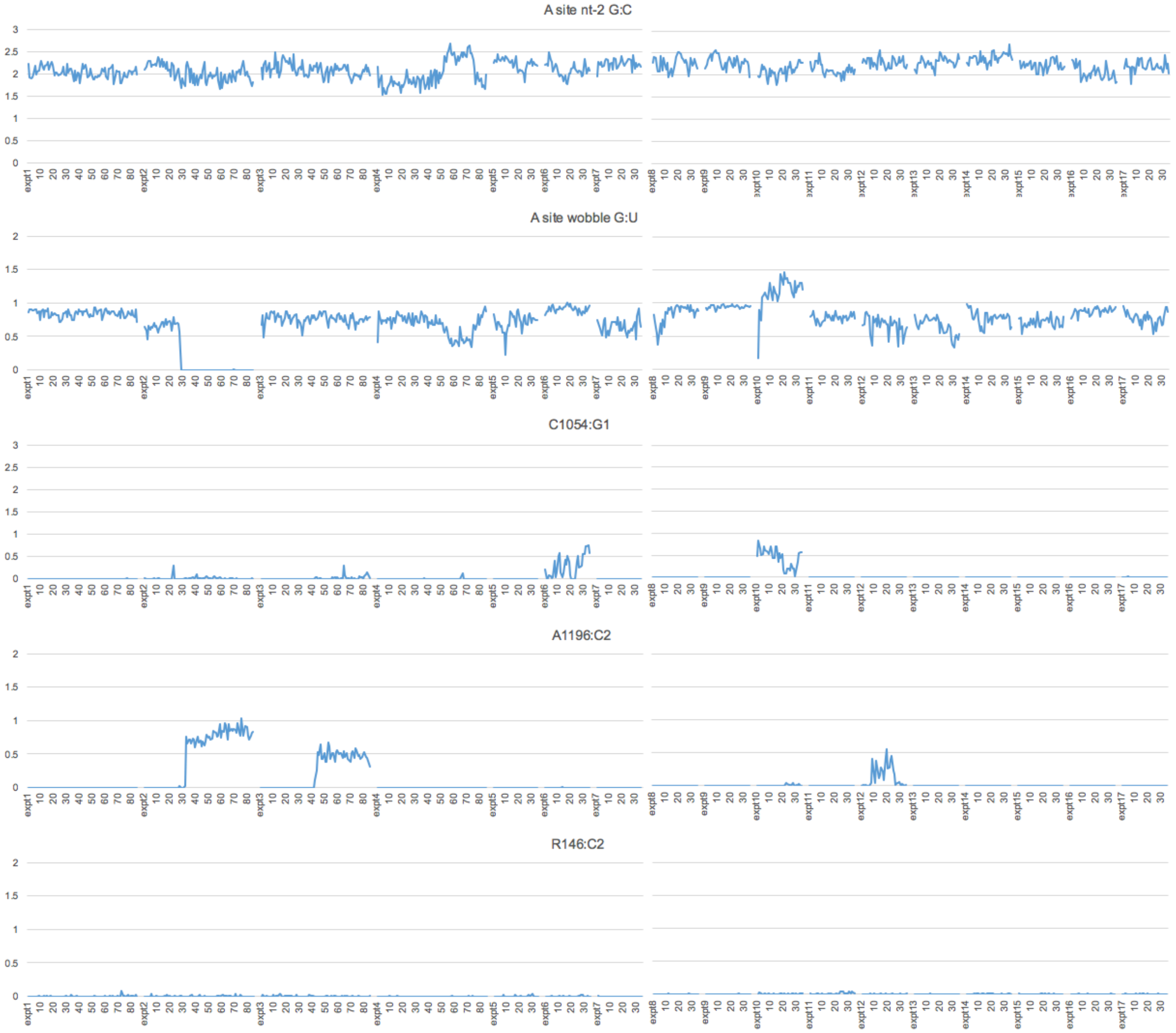

H-bonds over time (ns) cont.

Stage V

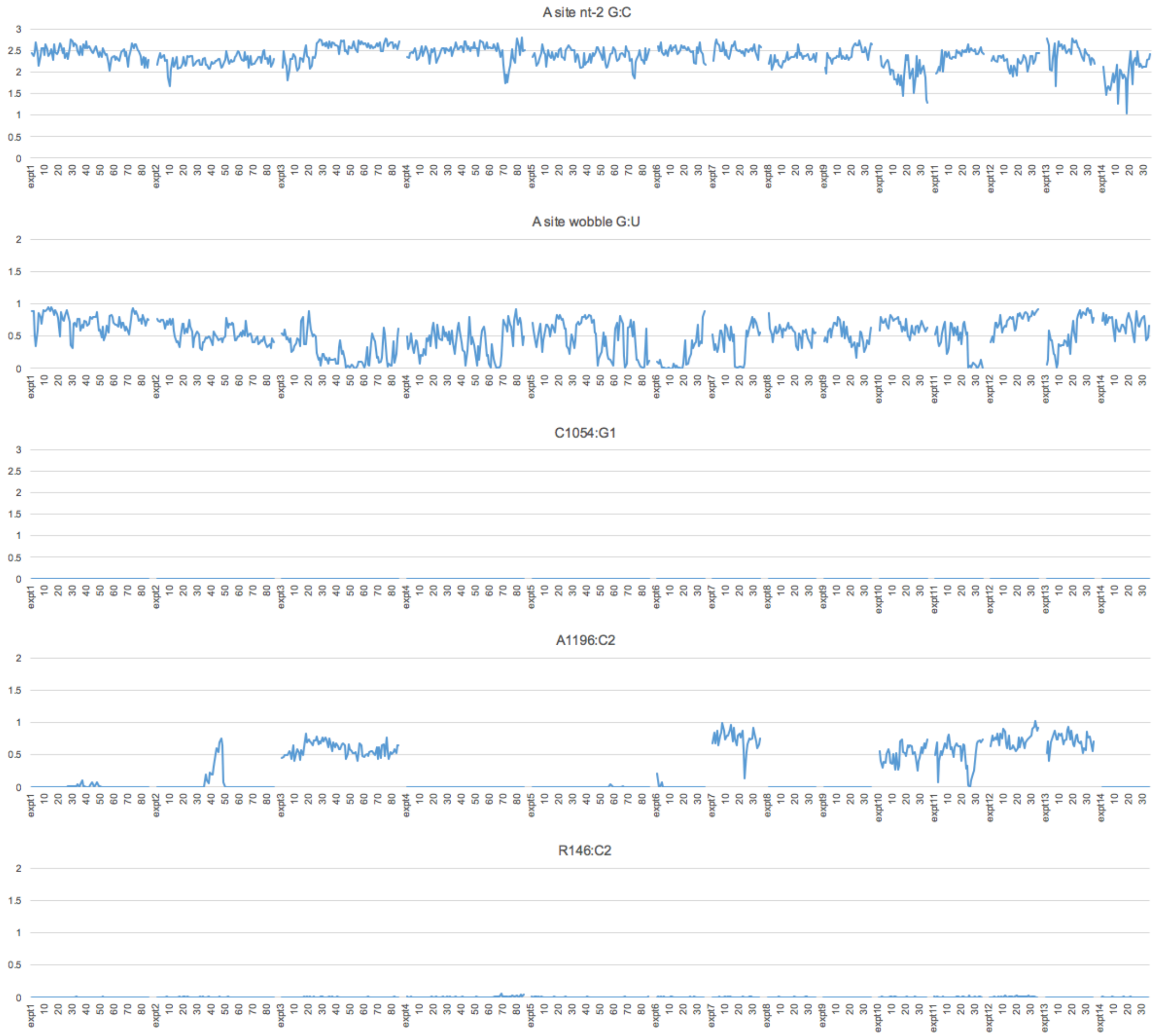

Figure S2. Numbers of H-bonds in MD replicate experiments. For each translocation stage (stages I through V), multiple independent MD experiments were run starting with different heat, random assignment of velocities, and equilibration. Numbers of H-bonds are between: A site nt-2 G:C; A site wobble base G:C; C1054:G1 of +1 codon; A1196 Hoogsteen edge:C2 Watson-Crick edge of +1 codon; R146 guanidinium group:C2 Watson-Crick edge. 100 frames were collected per ns of MD and the average number of H-bonds in each ns was plotted.
