# Supplementary material for: A Ribosome Interaction Surface Sensitive to mRNA GCN Periodicity
Source: Biomolecules. 2020 Jun 3;10(6):849. doi: 10.3390/biom10060849 (PMC7357141; doi:10.3390/biom10060849)
Supplement: Supplementary file 1 [file biomolecules-10-00849-s001.zip › supporting_final/FigS3.pdf]

Figure S3

Stacking distance (Å) over time (ns)

Stage I

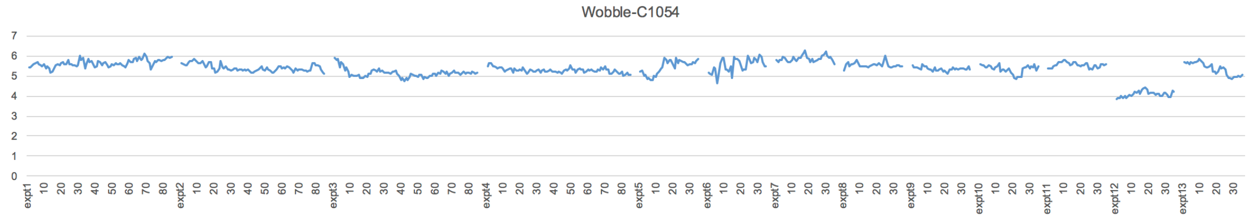

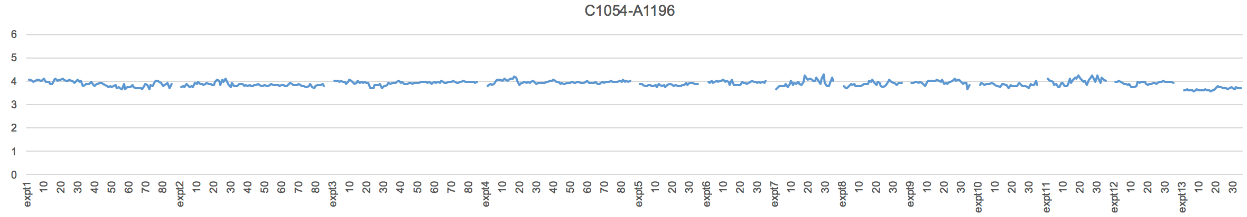

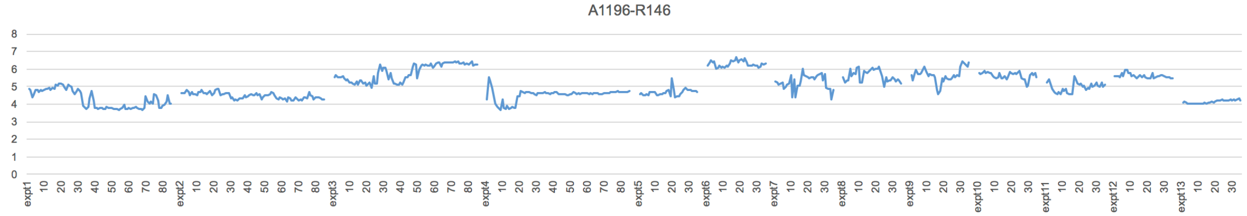

Stage II

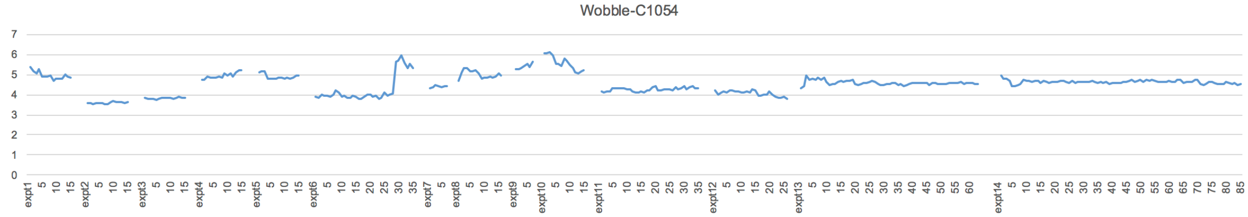

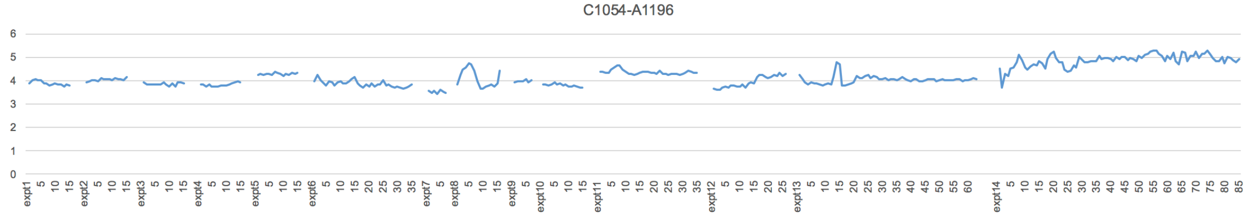

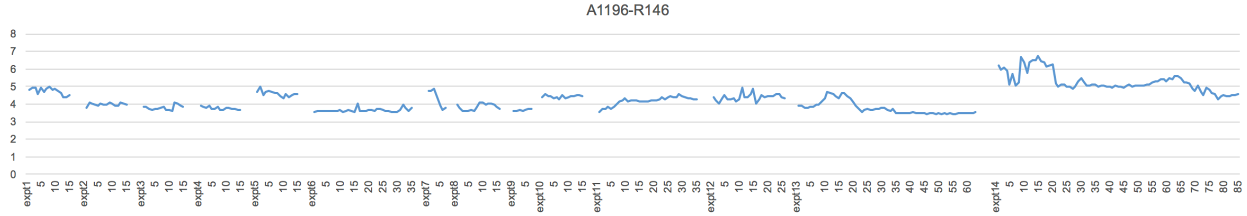

Stacking distance (Å) over time (ns) cont.

Stage III

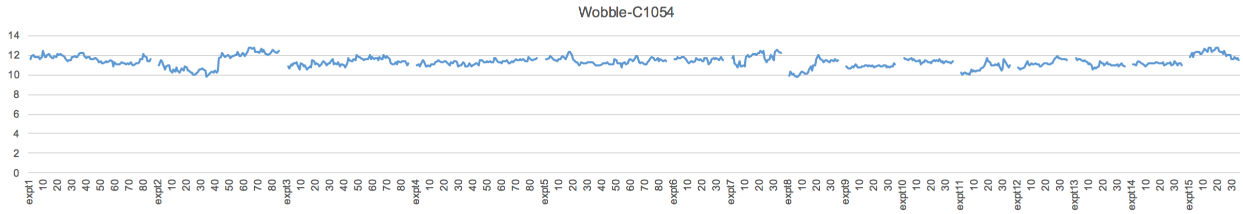

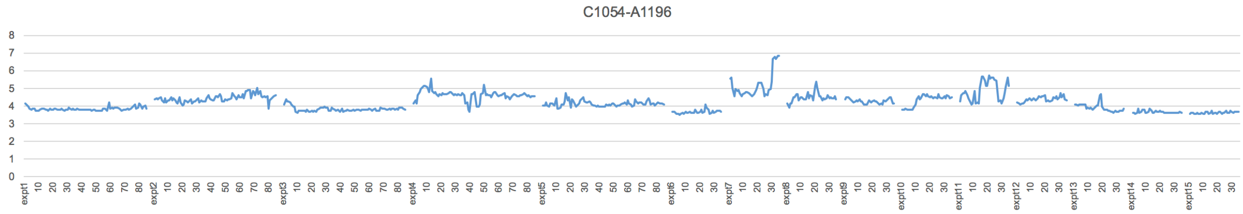

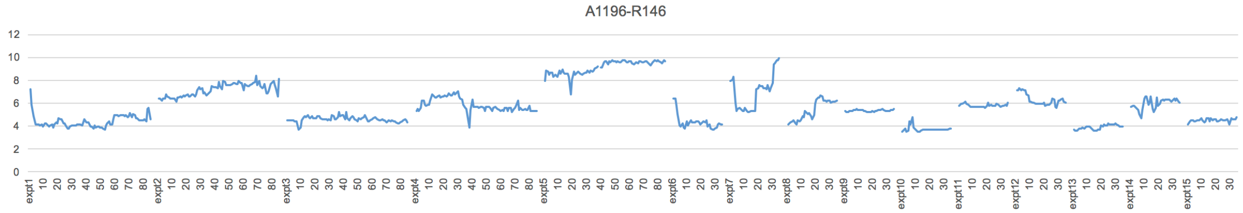

Stage IV

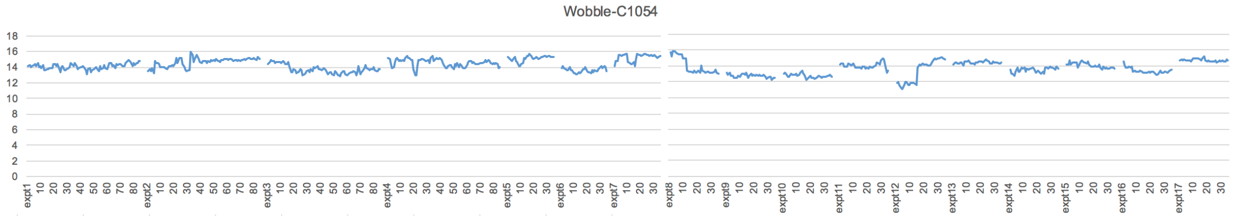

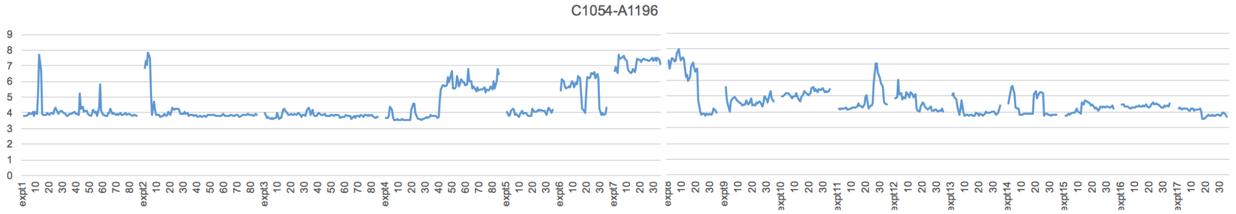

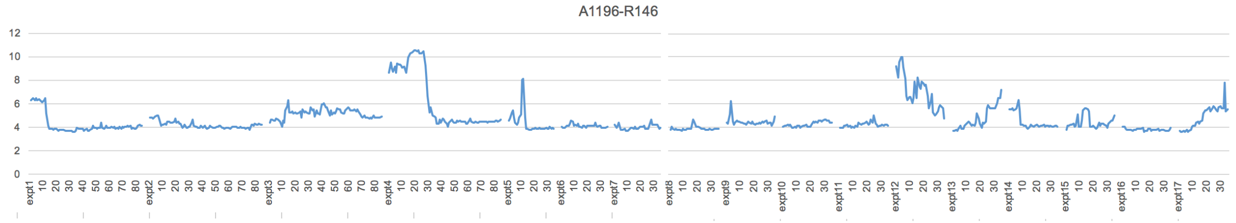

Stacking distance (Å) over time (ns) cont.

Stage V

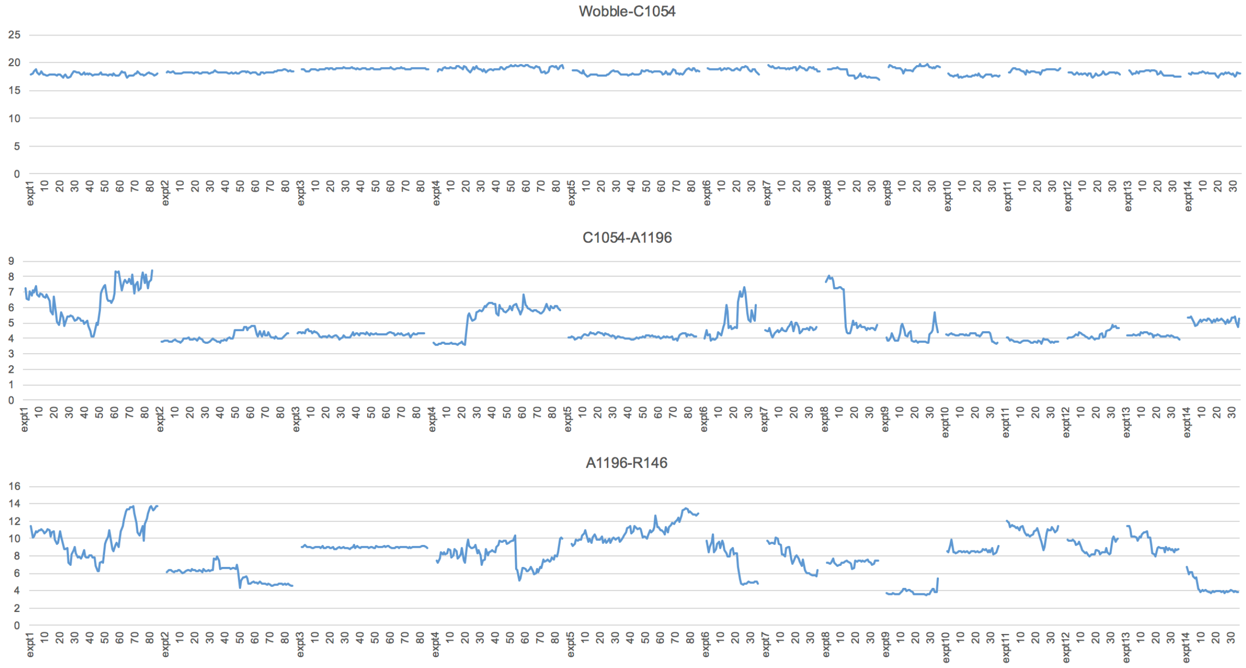

Figure S3. Stacking distances in MD replicate experiments. For each translocation stage (stages I through V), multiple independent MD experiments were run starting with different heat, random assignment of velocities, and equilibration. Distances (Å) are between the centers of masses of the bases: A site wobble base-C1054; C1054-A1196; A1196-R146 guanidinium group. Frames were pooled in 1-ns bins. 100 frames were collected per ns of MD and the average distance for each ns was plotted.
