# Supplementary material for: A Ribosome Interaction Surface Sensitive to mRNA GCN Periodicity
Source: Biomolecules. 2020 Jun 3;10(6):849. doi: 10.3390/biom10060849 (PMC7357141; doi:10.3390/biom10060849)
Supplement: Supplementary file 1 [file biomolecules-10-00849-s001.zip › supporting_final/FigS4.pdf]

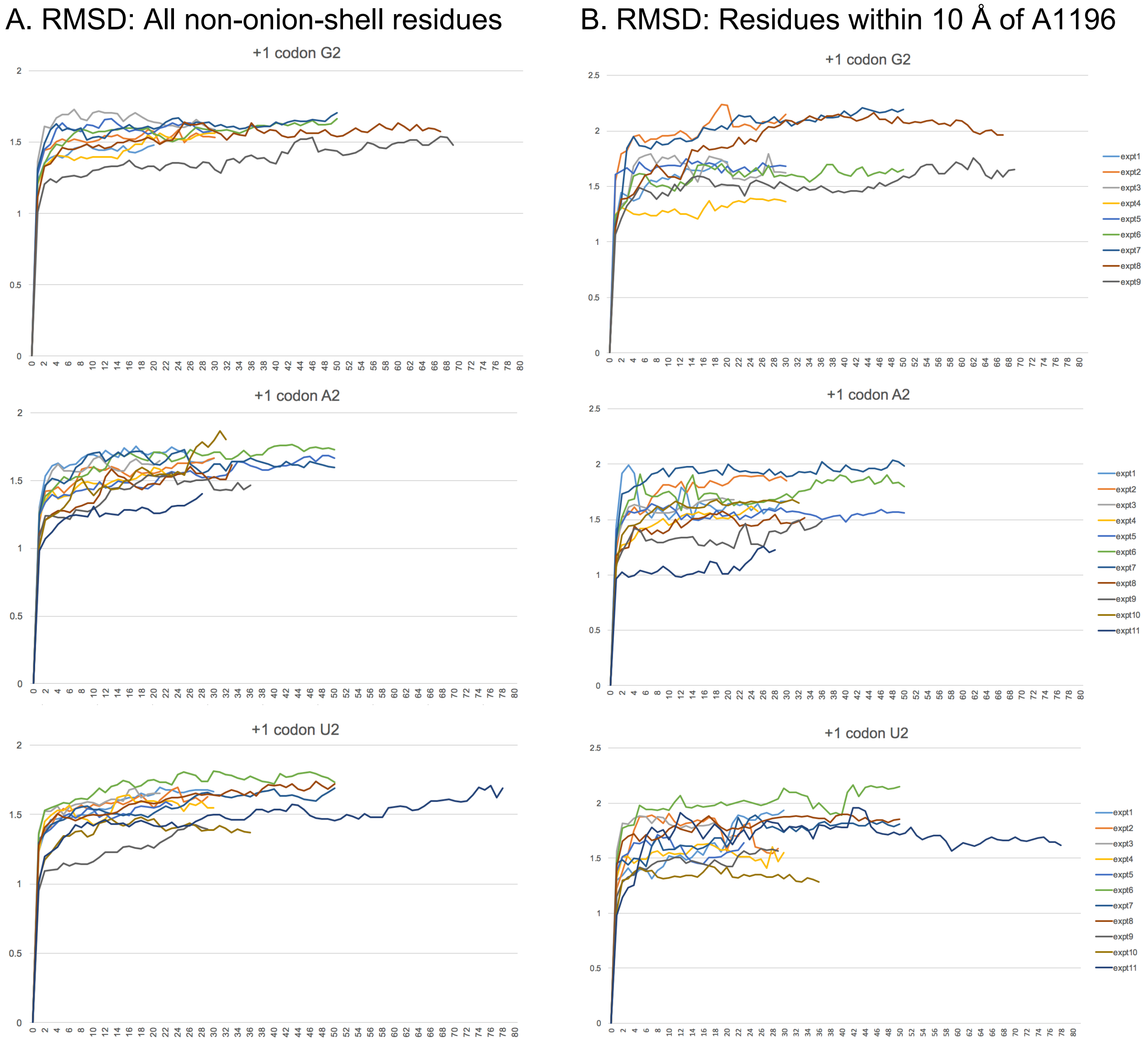

Figure S4. RMSD profiles for +1 codon nt-2 substitutions. C at position 2 of the +1 codon (C2) was replaced with G, A or U (G2, A2, U2) in the translocation stage II structure. (A) RMSD profiles for backbone atoms of all residues except those in restrained onion shell. RMSD profiles for multiple independent MD runs stabilized below 2 Å within 10 to 15 ns. The MD runs were analyzed starting at 15 ns. The RMSD profiles commence after 3 ns of equilibration during which backbone atoms of all residues were restrained at 20 kcal/mol Å^2. (B) RMSD profiles for backbone atoms of 19 residues located within 10 Å of A1196. MD runs stabilized below 2.5 Å within 10 to 15 ns.
