# Supplementary material for: A Ribosome Interaction Surface Sensitive to mRNA GCN Periodicity
Source: Biomolecules. 2020 Jun 3;10(6):849. doi: 10.3390/biom10060849 (PMC7357141; doi:10.3390/biom10060849)
Supplement: Supplementary file 1 [file biomolecules-10-00849-s001.zip › supporting_final/TableS1.pdf]

Table S1

Chain	5JUP Numbering*	5JUP Restrained	Subsystem Numbering	Subsystem Restrained
A 18S rRNA	1-31	1-5, 11-17, 22-31	1-31	1-5, 11-17, 22-31
A 18S rRNA	547-600	547-548, 550, 554-556, 588-600	32-85	32-33, 35, 39-41, 73-85
A 18S rRNA	1108-1113	1108-1113	86-91	86-91
A 18S rRNA	1133-1140	1133-1136, 1139-1140	92-99	92-95, 98-99
A 18S rRNA	1269-1279	1269-1270, 1277-1279	100-110	100-101, 108-110
A 18S rRNA	1424-1431	1424-1425, 1429-1431	111-118	111-112, 116-118
A 18S rRNA	1438-1442	1438-1442	119-123	119-123
A 18S rRNA	1629-1649	1629-1631, 1635-1637, 1644-1649	124-144	124-126, 130-132, 139-144
A 18S rRNA	1751-1763	1751-1752, 1759-1763	145-157	145-146, 153-157
A 18S rRNA	1780-1782	1780-1782	158-160	158-160
B 25S rRNA	2255-2258	2255-2256	161-164	161-162
EC mRNA (IRES)	6903-6909	6903, 6909	165-171	165, 171
EC mRNA (IRES)	6947-6958		172-183	
UB uS12 (yeast S23)	53-145	53-56, 73-84, 96-111, 121-134, 138-145	184-276	184-187, 204-215, 227-242, 252-265, 269-276
ZA uS5 (yeast S2)	85-98	85-98	277-290	277-290
ZA uS5 (yeast S2)	193-211	193-211	291-309	291-309
BC eS30 (yeast S30)	2-25	2-4, 22-25	310-333	310-312, 330-333
BC eS30 (yeast S30)	45-61	45-54, 61	334-350	334-343, 350
AB uS3 (Yeast S3)	111-117	111-117	351-357	351-357
AB uS3 (Yeast S3)	136-153	136-138, 150-153	358-375	358-360, 372-375
AB uS3 (Yeast S3)	176-180	176-177, 179-180	376-380	376-377, 379-380
DC yeast eEF2	578-588	578-588	381-391	381-391
DC yeast eEF2	608-711	608-660, 667-695, 710-711	392-495	392-444, 451-479, 494-495

*Same residue numbering for 5JUO, 5JUS, 5JUT, and 5JUU.
