# Supplementary material for: A Ribosome Interaction Surface Sensitive to mRNA GCN Periodicity
Source: Biomolecules. 2020 Jun 3;10(6):849. doi: 10.3390/biom10060849 (PMC7357141; doi:10.3390/biom10060849)
Supplement: Supplementary file 1 [file biomolecules-10-00849-s001.zip › supporting_final/TableS2.pdf]

Table S2

energy minimization round	harmonic restraint (kcal/mol Å^2)	steepest descent steps	conjugate gradient steps
1	100	2500	17500
2	75	2500	7500
3	65	2500	2500
4	55	2500	500
5	45	2500	500
6	30	2000	0
7	20	2000	0
8	15	2000	0
9	10	2000	0
10	5	2000	0
11	1	2000	0
